# Cancer Stem Cells in Intrahepatic Cholangiocarcinoma; Their Molecular Basis, and Therapeutic Implications

**DOI:** 10.3389/fphys.2021.824261

**Published:** 2022-01-17

**Authors:** Keiichi Tamai, Haruna Fujimori, Mai Mochizuki, Kennichi Satoh

**Affiliations:** ^1^Division of Cancer Stem Cells, Miyagi Cancer Center Research Institute, Natori, Japan; ^2^Division of Gastroenterology, Tohoku Medical and Pharmaceutical University, Sendai, Japan

**Keywords:** intrahepatic cholangiocarcinoma, cancer stem cells, mitochondria, dormant, epithelial-mesenchymal transition

## Abstract

Cancer tissue consists of heterogenous cell types, and cancer stem cells (CSCs) are a subpopulation of the tissue which possess therapy resistance, tumor reconstruction capability, and are responsible for metastasis. Intrahepatic cholangiocarcinoma (iCCA) is one of the most common type of liver cancer that is highly aggressive with poor prognosis. Since no target therapy is efficient in improving patient outcomes, new therapeutic approaches need to be developed. CSC is thought to be a promising therapeutic target because of its resistance to therapy. Accumulating evidences suggests that there are many factors (surface marker, stemness-related genes, etc.) and mechanisms (epithelial-mesenchymal transition, mitochondria activity, etc.) which are linked to CSC-like phenotypes. Nevertheless, limited studies are reported about the application of therapy using these mechanisms, suggesting that more precise understandings are still needed. In this review, we overview the molecular mechanisms which modulate CSC-like phenotypes, and discuss the future perspective for targeting CSC in iCCA.

## Introduction

The molecular mechanisms leading to cancer heterogeneity are still largely unknown, especially in cholangiocarcinoma, although its heterogeneity is known and was analyzed decades ago (Gray and Pierce, 1964; Fidler, 1978). A while back, a study regarding repopulating leukemia cells in NOD/SCID mice was published (Bonnet and Dick, 1997). Since then, there have been many papers about these repopulating cells, namely cancer stem cells (CSCs). A key concept in the field, that tumor tissue consists of several subpopulations, where each subpopulation has plasticity to transform the other, and removing a specific population can lead to shrinking of the whole tumor, significantly changes our understanding of cancer. Currently, CSCs are characterized by dormancy, chemotherapy resistance, altered mitochondrial activity, high proliferation, asymmetric division, in addition to self-renewal and differentiation capacity, which are common features observed in normal stem cells. Thus, the term CSC does not define a specific type of cell, but rather is a general term for therapy-resistant subpopulations of heterogenic cancer cells. These cells should be analyzed by focusing on specific phenotypes and underlying molecular mechanisms.

Intrahepatic cholangiocarcinoma (iCCA) is characterized as cholangiocarcinoma proximal to the second order bile ducts (Blechacz et al., 2011). Clinically, iCCA presents with unspecific symptoms and infiltrates easily into surrounding interstitial connective tissue, resulting in progressive disease and the loss of a chance to undergo surgery at the time of detection. Systemic therapy has been investigated and tried, but it is not easy to improve the patient’s prognosis.

## Possible Presence of Stem Cells in Intrahepatic Cholangiocarcinoma

It is widely accepted that the subset of cells with stem and progenitor characteristics in normal tissues are particularly susceptible to oncogenic transformation (Lytle et al., 2018). Tomasetti and Vogelstein (2015) reported that the lifetime risk of cancers is strongly correlated with the total number of divisions of the normal self-renewing cells maintaining that tissue’s homeostasis, indicating that cancer cells originate from normal stem cells. The cell of origin for cancers is known to be closely associated with the nature of the cancer (Lytle et al., 2018). For example, while BCR-ABL rapidly triggered chronic myeloid leukemia (CML) when introduced into stem cells, it triggered B cell acute lymphocytic leukemia (ALL) when expressed in progenitor cells. In iCCA, there are two main different histological subtypes which originated from different cell types (Bragazzi et al., 2018). The large bile duct (mucinous) type iCCAs arise in larger intrahepatic bile ducts, and the small bile duct (mixed) type iCCAs show a profile similar to mucin-negative cuboidal cholangiocytes that line the smaller bile duct (interlobular bile duct and bile ductules).

In the normal liver, intrahepatic biliary epithelium can proliferate in a liver injury model, but proliferative biliary epithelial cells depend on a stochastically maintained progenitor population (Kamimoto et al., 2016). In cholangiocarcinoma, the presence of CSCs has been speculated in pathological observations made decades ago. In a choline-deficient and acetylaminofluorene fed rats, oval cells (stem cells of liver) proliferate and cholangiocarcinoma was observed (Sell and Dunsford, 1989). Ishikawa et al. reported a case of iCCA producing α-fetoprotein (AFP), which is usually produced in liver cancer and normal fetal liver (Ishikawa et al., 2007). In immunohistochemistry, AFP, cytokeratin (CK)7 (biliary epithelial marker), CK14 (a liver stem cell marker), and CD133 (hematopoietic and CSC marker) were colocalized, and the authors concluded that the tumor was derived from a normal liver stem cell. Transcriptomic profiling of clinical tissues suggested that gene expression signatures of iCCA shared the signatures of hepatocellular carcinoma (HCC) with stem cell gene expression traits (Oishi et al., 2012). These data indicate that iCCA arise from liver stem cells and exhibit stem-like capacity. However, whether stem cells in cancer initiation stage and CSCs in the cancer progression stage are the same subset of cells is still under discussion.

## Surface Markers Specific for Cancer Stem Cells

Since the CSCs are identified as CD34^+^CD38^–^ population (Bonnet and Dick, 1997), cell surface proteins have been intensively explored as CSCs marker (Najafi et al., 2019). Many of CSCs markers are also known as normal stem cell markers [CD133 (Barzegar Behrooz et al., 2019), CD44 (Morath et al., 2016), LGR5 (Shimokawa et al., 2017), etc.]. In iCCA, CD133, and CD44 are suggested to be CSC markers since they were correlated with the CSC like features as described below.

CD133, known as prominin-1 (a transmembrane glycoprotein), is widely acknowledged as a CSC marker (Barzegar Behrooz et al., 2019). CD133-positive cases are related to a poor prognosis in iCCA (Shimada et al., 2010). CD133 also correlates with poor prognosis in extrahepatic bile duct cancer (Mizukami et al., 2018). Although CD133 in cholangiocarcinoma correlates with tumor malignancy, the pathophysiological roles of CD133 are still unknown. In Caco-2 (colorectal cancer) and OVCAR-8 (ovarian cancer) cells, CD133 binds Histone Deacetylase 6 (HDAC6) and CD133 is degraded by lysosomes (Mak et al., 2012). CD133/HDAC6/β-catenin complex is stable and increases cell proliferation and *in vivo* tumorigenicity.

CD44 belongs to the family of non-kinase, single span, transmembrane glycoproteins expressed on embryonic stem cells (Chen et al., 2018). Many isoforms with slightly different functions are transcribed from the CD44 gene. In gastric cancers, CD44v8-10 interacts with glutamate-cysteine transporter xCT and increases cystine transport and GSH synthesis, resulting in suppression of reactive oxygen species (ROS) and p38 activation (Ishimoto et al., 2011), indicating that CD44 variants are involved in supporting the functions of CSC’s in gastric cancer. Knock down of CD44v9 in an iCCA cell line caused the decrease of tumor growth *in vivo* and in epithelial mesenchymal transition (EMT). Sulfasalazine is a xCT inhibitor (Liu et al., 2020) which inhibits cell proliferation and induces cell death through decrease of glutathione and increase of ROS, indicating the possibility of iCCA therapy (Thanee et al., 2016).

## Epithelial Mesenchymal Transition in Cancer Stem Cells

Epithelial mesenchymal transition has been recognized as an indispensable mechanism during embryogenesis (Nieto et al., 2016). The EMT process also confers malignant traits, such as motility, invasiveness, and resistance to apoptosis in neoplastic cells (Peinado et al., 2007). In addition, EMT induces stem cell properties in epithelial neoplasms (Mani et al., 2008). ZEB1 is a transcription factor that promotes metastatic and stem cell features (Oishi et al., 2012; Pastushenko and Blanpain, 2019). ZEB1 induces pluripotent factors such as SOX2, NANOG, and OCT4 through miR-200 in prostate cancer cells (Kong et al., 2010). In iCCA, ZEB1 is expressed both in tumor and stroma, and plays a key role in the induction of EMT in tumor cells (Lobe et al., 2021). Expression of ZEB1 in tumor cells leads to induction of EMT and stemness, and functions at both sides of the tumor-stroma interface by regulating the production of connective tissue growth factor (CTGF), hepatocyte growth factor (HGF), and interleukin 6 (IL6), to boost tumor progression. TGF-β is a strong EMT inducer and caused increase aldehyde dehydrogenase (ALDH) activity and resistance to 5-FU treatment in a iCCA cell line (Shuang et al., 2014); thus, suggesting a close relationship between EMT and CSC.

## Dormant Cancer Stem Cells in Intrahepatic Cholangiocarcinoma

Cancer stem cells are recognized as therapy-resistant cells, especially the resistance to chemotherapy or radiation (Batlle and Clevers, 2017). These therapies are more effective against proliferative cells. Thus, CSCs might be dormant or quiescent. Dormant cancer cells are in pathological state when compared with quiescent cells, and can be in a state of temporary mitotic arrest over prolonged periods (Hadfield, 1954; Phan and Croucher, 2020). Dormant cancer cells are recognized as therapy-resistance cells and as a cause for long-term recurrence of cancer. Continued exploration into the underlying biology of tumor dormancy is critical to advance the development of more effective therapies that prevent and/or treat metastasis to ultimately improve patient survival.

Tumor dormancy and its regulators have been investigated in some types of cancers. DYRK1B is a kinase which regulates G_0_/G_1_-S phase transition in cancer (Becker, 2018). Knockdown of DYRK1B reduced quiescent cells and increased gemcitabine sensitivity in pancreatic cancer cell lines (Ewton et al., 2011). In a mouse model of CML, ablation of Fbxw7 induced long-term stem cells to enter the cell cycle, and Fbxw7-deficient leukemia stem cells were sensitized to Ara-C and imatinib (Takeishi et al., 2013).

In liver cancer, several studies exploring dormant CSCs have been reported. CD13, also known as aminopeptidase N, is a ubiquitous transmembrane ectoenzyme (Amin et al., 2018). It is found mainly in the liver, brush border of the kidneys, small intestine, and placenta. In HCC, CD13 is a marker for semiquiescent CSCs (Haraguchi et al., 2010). CD13^+^CD90^–^ CSCs are dormant and exhibit reduced intracellular ROS levels, whereas CD13^–^CD90^+^ CSCs actively proliferate and are sensitive to therapy. In cholangiocarcinoma, CD274^low^ (also known as PD-L1) cancer cells are dormant and possess high tumorigenicity (Tamai et al., 2014). In CD274^low^ cells, brain expressed gene 2 (*BEX2*) is highly expressed, and BEX2^high^ cells are dormant CSCs in iCCA and HCC (Tamai et al., 2020; Fukushi et al., 2021). Knockdown of BEX2 induced a proliferating phase in the cell cycle in iCCA cell lines. BEX2 was also highly expressed in hepatoblasts and decreased in normal bile ducts of mice, suggesting the crucial role in normal stem cells.

## Mitochondria in Cancer Stem Cells

Mitochondria perform an essential function in cells by coordinating both the production and distribution of energy by oxidative phosphorylation based on oxygen and substrate availability. In recent years, mitochondria have been shown to be key players in the maintenance of stem cells. The selective degradation of mitochondria, or mitophagy, has been directly implicated in stem cell self-renewal (Katajisto et al., 2015). In hematopoietic stem cells, low mitochondrial membrane potential marks self-renewing hematopoietic stem cells (Sukumar et al., 2016). In mice models of pancreatic cancer, inhibition of PGC1A, a master regulator of mitochondrial biogenesis, reduced organoid growth (Nimmakayala et al., 2021). In hepatic CSCs, mitophagy is also a critical step to maintain cancer stemness through p53 phosphorylation and NANOG expression (Liu et al., 2017). Degradation of mitochondria leads to the reduction of mitochondrial mass and respiration, which are proposed to contribute to the quiescent state of stem cells, and the maintenance of the quality of mitochondria. Contrarily, increased mitochondrial biogenesis is usually connected with a higher tumorigenic rate, which is a hallmark of CSC’s phenotype (Farnie et al., 2015). In iCCA, oxygen consumption ratio was increased in spheroid culture than monolayer culture (Raggi et al., 2021). Knockdown of PGC1A caused the reduction of spheroid formation. BEX2 suppresses mitochondrial activity through mitochondrial protein TUFM, resulting in the induction of dormant phase because of the reduction of mitochondrial respiration and increase tumorigenicity (Tamai et al., 2020). It would be important to distinguish the characters of dormancy and tumorigenicity, both of which are known as characteristics of CSC’s, and analyze the underlying pathways.

## Application to Diagnosis and Treatment

Several anti-cancer therapies targeting CSCs were proposed and initiated by pharmaceutical companies to treat various types of cancers (Batlle and Clevers, 2017). However, studies on potential inhibitors against CSCs to effectively treat iCCA have been extremely limited.

Doublecortin-like kinase 1 (DCLK1) is a protein associated with microtubules in cytoplasm which catalyzes the polymerization of microtubules (Westphalen et al., 2017). DCLK1 was found in CSCs of gastrointestinal tract tumors such as colon (Chandrakesan et al., 2017), pancreas (Westphalen et al., 2016), and hepatocarcinoma (Ali et al., 2015). Serum concentration of DCLK1 was higher in iCCA and perihilar CCA (pCCA), and lower in healthy and primary sclerosing cholangitis (PSC) patients (Lorenzo et al., 2021). This is the first study of diagnostic tools using a CSC marker, although the mechanism of DCLK1 secretion specific to CCA have not been described. Recently, several micorRNAs and long non-coding RNAs have been reported to be useful for iCCA diagnosis (Zheng et al., 2017), and the role of non-coding RNAs in the stemness of cholangiocarcinoma gradually become evident (Gao et al., 2020; Lu et al., 2021).

In cholangiocarcinoma cell lines, gemcitabine-resistant cells show CSC-like phenotype, such as high ALDH activity and high CD44 expression (Kawamoto et al., 2018). Metronidazole is effective for gemcitabine-resistant cells but not normal cells, although the underlying mechanism is not clear.

Yes-associated protein (YAP) is a major downstream effector of the Hippo signaling pathway and maintains CSC-like property. YAP-positive cases demonstrated poor prognosis in iCCA (Sugiura et al., 2019). The knockdown of YAP decreases the CD133 expression and verteporfin, a YAP inhibitor decreased sphere formation and tumorigenicity.

The Sonic Hedgehog (SHH) pathway plays a pivotal role in various human malignant neoplasms. Under a hypoxic environment, the SHH pathway was promoted in iCCA cell lines and the CSC-related genes including NANOG, Oct4, SOX2, and CD133 were upregulated (Bhuria et al., 2019). Cyclopamine, a SHH inhibitor, suppressed a hypoxia induced SHH pathway and decreased invasion capacity.

Brain expressed gene 2, described above, is a key player of dormant CSCs in liver cancer, and a small compound 1,3-Benzenediol, 4-[4-(4-methoxyphenyl)-1H-pyrazol-3-yl] (BMPP) can promote BEX2 degradation, and the treatment with BMPP induces cell proliferation (Saijoh et al., 2021). Under BMPP treatment, cisplatin and gemcitabine sensitivity are increased in hepatocellular and cholangiocarcinoma cell lines.

## Conclusion

In this work, we review the recent progress of CSC research in iCCA (Table 1). The therapeutic approach against dormant cancer cells in cholangiocarcinoma is promising, but a specific inhibitor against CSCs is hard to develop at present. Even though iCCA exhibits aggressive malignancy, the detailed underlying mechanisms are not yet fully understood. Going forward, the crosstalk between immune cells and CSCs, and alternative metabolic pathway in CSCs should be investigated.

**TABLE 1:** Important proteins for the functioning of the cancer stem cells (CSCs) of intrahepatic cholangiocarcinoma (iCCA).

Molecule	Function	References
CD133	Binds HDAC6, but function almost unknown	Shimada et al., 2010
CD44	Binds xCT and increases cystine transport and GSH synthesis	Ishimoto et al., 2011; Thanee et al., 2016
ZEB1	Induces EMT and stemness	Lobe et al., 2021
TGF-β	EMT inducer; increases ALDH activity and resistance to 5-FU	Shuang et al., 2014
BEX2	Induces dormancy	Tamai et al., 2020
PGC-1a	Regulator of mitochondrial biogenesis	Raggi et al., 2021
DCLK1	Catalyzes microtubule polymerization	Lorenzo et al., 2021
YAP	Increases sphere formation and tumorigenicity	Sugiura et al., 2019
SHH	Increases invasiveness under hypoxic condition	Bhuria et al., 2019

## Author Contributions

All authors listed wrote, edited, and revised the manuscript and approved it for publication.

## Conflict of Interest

The authors declare that the research was conducted in the absence of any commercial or financial relationships that could be construed as a potential conflict of interest.

## Publisher’s Note

All claims expressed in this article are solely those of the authors and do not necessarily represent those of their affiliated organizations, or those of the publisher, the editors and the reviewers. Any product that may be evaluated in this article, or claim that may be made by its manufacturer, is not guaranteed or endorsed by the publisher.
